# Paraprotein interference in bilirubin assays: A case of multiple myeloma highlighting the reliability of dry chemistry

**DOI:** 10.1177/00045632261435377

**Published:** 2026-03-16

**Authors:** Aditya Narayanan S, Ramya Devi D A, Renuka Pangaluri, Vinodhini V M, Arul Senghor K A

**Affiliations:** Department of Biochemistry, Faculty of Medicine and Health Sciences, SRM Institute of Science and Technology, 29945 SRM Medical College Hospital and Research Centre, Kattankulathur, Tamil Nadu, India

**Keywords:** Multiple myeloma, dry chemistry, wet chemistry, paraprotein interference, negative direct bilirubin

## Abstract

The presence of paraproteins in multiple myeloma can cause analytical interference, resulting in unusual and misleading biochemical outcomes. An uncommon but clinically relevant finding is the reporting of a negative direct bilirubin result on wet chemistry analyzers—an impossible outcome that strongly suggests analytical interference. A 60-year-old male presented with nonspecific symptoms. Liver function tests carried out on a wet chemistry analyzer indicated a total bilirubin of 0.39 mg/dL and a direct bilirubin of −7.67 mg/dL, which was not physiologically possible. There was no evidence of jaundice, and imaging appeared normal. Repeat testing with a dry chemistry analyzer indicated a total bilirubin of 1.0 mg/dL and a direct bilirubin of 0.1 mg/dL, aligning with the clinical picture. Further investigations confirmed the diagnosis of multiple myeloma with IgG-kappa monoclonal gammopathy. The discrepancy was attributed to paraprotein interference in the wet chemistry method. This case highlights a rare but important laboratory artifact—negative direct bilirubin due to paraprotein interference—and emphasizes the reliability of dry chemistry in such scenarios. Awareness of this interference is important for accurate diagnosis and avoiding unnecessary workup.

## Introduction

Multiple myeloma is a plasma cell malignancy characterized by the overproduction of monoclonal immunoglobulins, often leading to high circulating protein levels. These paraproteins can interfere with routine biochemical assays, especially those performed using wet chemistry systems.

While paraprotein-related interference is well recognized in tests like sodium, calcium, and albumin, its effect on bilirubin estimation is less commonly reported. Bilirubin measurement, especially using diazo-based reactions in wet chemistry analyzers, can be affected by protein turbidity and precipitation. This case illustrates such interference and highlights the utility of dry chemistry systems in resolving the discrepancy.

## Case presentation

A 60-year-old male presented with chest pain, heart burn, and progressive fatigue over two months. He had no history of liver disease, hemolysis, or drug intake. On examination, pallor was present and there were no signs of jaundice and organomegaly.

**Table 1.** Analytical Results Obtained by Wet Chemistry and Dry Chemistry Platforms.

Test	Method	Result	Unit	Reference range
Initial Laboratory Workup (Wet Chemistry Platform – Beckman Coulter AU480)				
Total bilirubin	DPD/Jendrassik	0.39	mg/dl	0.5–1.0
Direct bilirubin	DPD/end point	−7.67	mg/dl	<0.3
Total protein	Biuret	13.9	g/dl	6.6–8.3
Albumin	Bromocresol green	1.7	g/dl	3.5–5.2
Globulin	Calculation	12.2	g/dl	2.5–3.0
Albumin/globulin ratio	Calculation	0.1		1.4–1.7
SGOT (AST)	UV Kinetic/IFCC	19	IU/L	<35
SGOT (ALT)	UV Kinetic/IFCC	10	IU/L	<45
Alkaline phosphatase	PNP AMP Kinetic	33	IU/L	30–120
Gamma GT	Kinetic/IFCC	9	U/L	<55
Repeat testing (dry chemistry platform – Quidel Ortho Vitros 5600):				
Total bilirubin	Azobilirubin/dyphylline	1.0	mg/dl	0.2–1.3
Direct bilirubin	Dual wavelength spectrophotometric	0.1	mg/dl	<0.3

The negative direct bilirubin level in the wet chemistry platform raised concern about potential analytical problems (Table 1). The reaction absorbance curve showing a non-linear pattern as in Figure 1(b) raised a suspicion of analytical interference. So, repeat testing was done using dry chemistry platform, in which the values were within normal limits, correlating with the clinical scenario. Paraprotein interference was suspected due to discrepancy.

**Figure 1.**
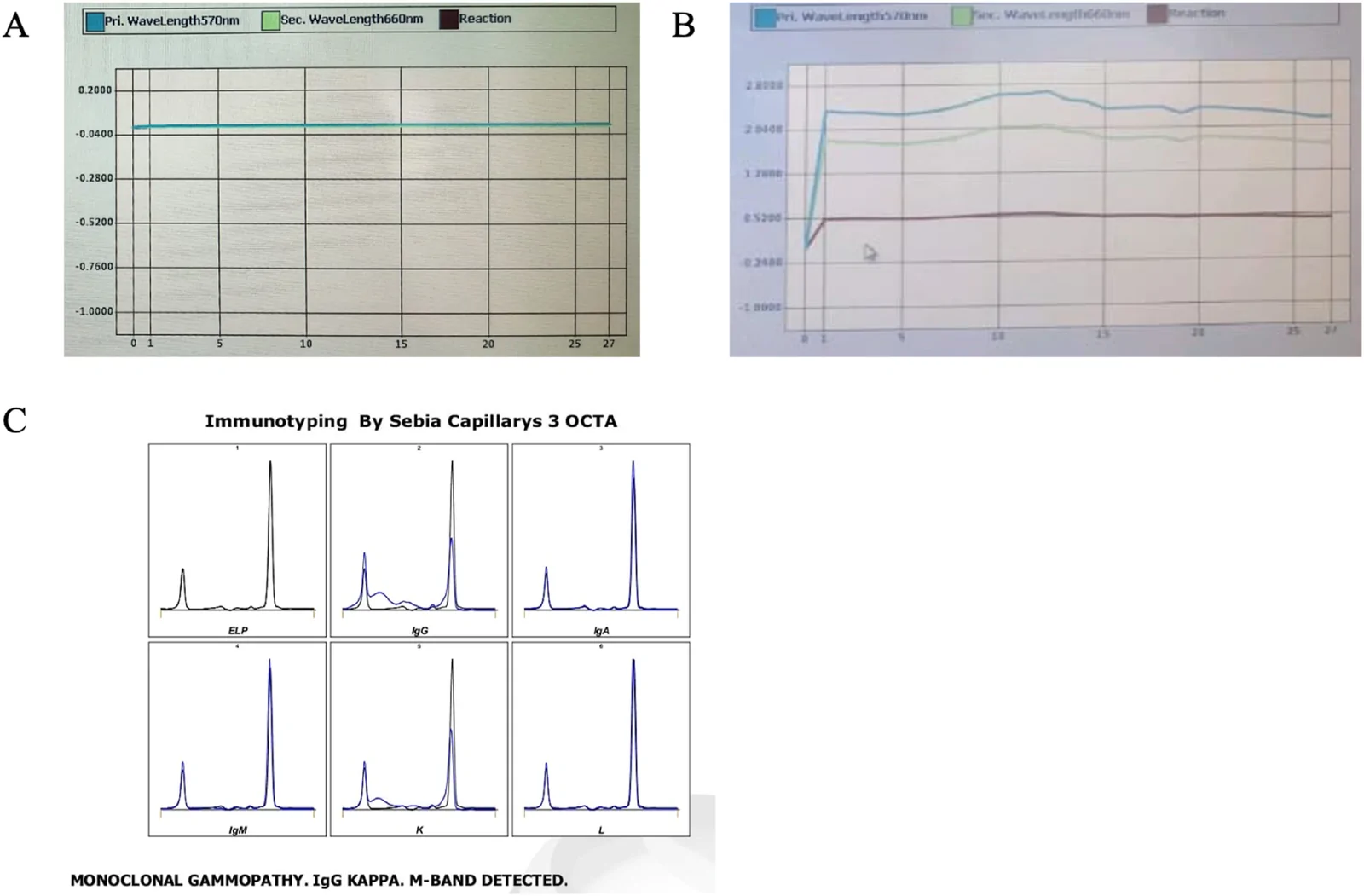
(a) Normal reaction absorbance curve of direct bilirubin on wet chemistry platform. (b) Erratic reaction absorbance curve of direct bilirubin on wet chemistry platform indicating analytical interference. (c) Abnormal immunoelectrophoresis (immunosubtraction) pattern indicating monoclonal gammopathy.

### Further workup

Immuno Electrophoresis (Immunosubtraction) (Figure 1(c)):

IgA—0.69 g/L (0.7–4)

IgG—74.30 g/L (5.49–15.84)

IgM—< 0.242 g/L (0.4–2.3)

Kappa Light Chain—20.30 g/L (1.38–3.75)

Lamba Light Chain—0.52 g/L (0.93–2.42)

Kappa Chain Lambda Chain Ratio—39.0385 (1.17–2.93).

## Diagnosis

Multiple myeloma with paraprotein-induced interference in bilirubin estimation was diagnosed.

## Discussion

Multiple myeloma is a neoplastic plasma cell disorder characterized by the clonal proliferation of malignant plasma cells in the bone marrow, resulting in overproduction of monoclonal immunoglobulins, known as paraproteins or M-proteins. These paraproteins can accumulate in various tissues causing hypercalcemia, renal insufficiency, anemia, osteolytic lesions, and eventually resulting in end-organ damage.^1^

Paraproteinemia refers to the presence of circulating monoclonal immunoglobulins, which is a hallmark of monoclonal gammopathies. Monoclonal gammopathies are group of disorders characterized by proliferation of single clone of plasma cells producing a monoclonal immunoglobulin (M-protein), with conditions ranging from benign to malignant plasma cell neoplasms.^2^ The commonly associated disorders include multiple myeloma, cryoglobulinemia, lymphoma, amyloidosis, Waldenstrom macroglobulinemia, and POEMS (Polyneuropathy, Organomegaly, Endocrinopathy, M-protein spike, and Skin manifestations).^3^ Paraprotein interference denotes the analytical errors that these proteins can cause in laboratory tests, mainly in assays that rely on turbidimetric, nephelometric, spectrophotometric, or chemical reactions.^4^ The commonly affected analytes by paraproteinemia include bilirubin (both total and direct), phosphate, iron, HDL-cholesterol, and unsaturated iron-binding capacity (UIBC).^5^ Such interference can falsely increase or decrease measured values, leading to missed diagnosis, misdiagnosis, mismanagement, or even iatrogenic morbidity and mortality.^6^

In this case, the patient initially presented with non-specific symptoms. The patient underwent routine laboratory investigations including liver function tests, wherein the direct bilirubin was found to be a negative value, an unlikely result which is physiologically impossible. The profile of the reaction curve on the wet chemistry analyzer made this result more suspicious. The curve showed an erratic, non-linear pattern, raising suspicion of analytical interference.^4^

The patient’s laboratory results showed significantly increased total protein and globulin fractions, with reversal of albumin-to-globulin ratio. This biochemical pattern is usually the initial indication of paraproteinemia. In estimation of bilirubin using wet chemistry platforms, this interference has been believed to be due to matrix effects—alterations in analyte detection due to the presence of interfering substances.^7^ The tendency of paraproteins to polymerize and precipitate may also compromise the assay’s precision.^7,8^ There are several other mechanisms for paraprotein interference, such as binding to the analyte, hyper viscosity, volume displacement, prozone effect, hook effect, and cryoglobulinemia. However, precipitation remains the most common cause of interference resulting in spuriously high or low values, frequently in combination with abnormal reaction curve.^4^

The specific analytical mechanisms of paraprotein interference due to precipitation involve the following factors: **(a) Acidic reaction environment:** Direct bilirubin assays typically occur in a strongly acidic medium. When the serum is mixed with these acidic reagents, the M-proteins precipitate, creating transient turbidity. **(b) Optical interference and light scattering:** The aggregated or precipitated proteins suspended in the reaction solution scatter light, which interferes with absorbance measurements. This interference often manifests as peculiar spikes in the reaction’s absorbance curve. **(c) Failure of stabilizing agents:** Most clinical chemistry reagents contain protein stabilizing agents designed to prevent precipitation. However, the solubilization capacity of these agents is often insufficient when M-protein concentrations are significantly higher than that of the usual concentrations of serum proteins.^4^

The precipitation of paraproteins results in increased turbidity of the sample. Since the absorbance of the sample blank cuvette is subtracted from the absorbance of the reaction cuvette, the absorbance may appear higher or lower due to variations in turbidity between the cuvettes. This explains the reason for the negative direct bilirubin value in this patient.^9^

The suspicion was strengthened when serum protein electrophoresis revealed a clear M-band, and follow-up immunofixation (immunosubtraction) showed a monoclonal gammopathy of IgG kappa type. Thus, the presence of paraproteins in the patient’s serum explained the abnormal bilirubin value.

Simple methods that can be employed to eliminate paraprotein interference in bilirubin assays are **(a) Sample Dilution:** This method involves diluting the patient’s serum to reduce the concentration of the interfering paraproteins. While dilution with normal saline is common, it may produce unsatisfactory results for some measurands or fail to normalize irregular reaction curves. A more effective approach is dilution with a healthy serum of known concentration, which maintains the original specimen matrix and antigen antibody reaction milieu.^6^ **(b) Physical Removal of Paraproteins:** Interfering proteins can be removed from the sample through ultrafiltration and de-proteinization.^4^ (c) **Chemical Treatment:** The sample can be treated with polyethylene glycol to precipitate the interfering proteins, allowing the supernatant to be retested for chemistry measurands. However, this method is often unsuitable for immunoassays because it may precipitate the protein intended for measurement.^4,6^ Additionally, 2-mercaptoethanol, a reducing agent that breaks down disulfide bonds in protein precipitates, can be used to dissolve paraprotein precipitates.^4^

To resolve the diagnostic uncertainty in this patient, bilirubin estimation was repeated using a dry chemistry platform. Dry chemistry makes use of multilayer slides with specific functional layers. The filter and spreading layers serve as membranes that divide large interfering proteins from the reagent zone and permit only the analyte of interest to react in the detection layer.^7^ Dry chemistry platform gave bilirubin values consistent with the clinical presentation, thus verifying both the analytical interference in wet chemistry and the accuracy of dry chemistry in such contexts.

This case highlights a key rule of clinical laboratory medicine: whenever laboratory results do not correlate with the clinical presentation, there is a possibility of analytical interference. Paraproteinemia, seen in multiple myeloma, is a cause for such discrepancy. Availability of alternative platforms like dry chemistry is a ready solution, with precise measurement and avoidance of misinterpretation.
